# Supplementary material for: Pharmacoeconomic Profiles of Advanced Therapy Medicinal Products in Rare Diseases: A Systematic Review
Source: Healthcare (Basel). 2025 Aug 2;13(15):1894. doi: 10.3390/healthcare13151894 (PMC12345686; doi:10.3390/healthcare13151894)
Supplement: Supplementary file 1 [file healthcare-13-01894-s001.zip › healthcare-3737520-supplementary.pdf]

Supplementary Materials

Pharmacoeconomic Profiles of Advanced Therapy Medicinal Products in Rare Diseases: A Systematic Review

Journal: **Healthcare MDPI**

Special Issue: **Healthcare Economics, Management, and Innovation for Health Systems**

Authorship
Marianna Serino [1], Milana Krstin [1], Sara Mucherino [1,2,*], Enrica Menditto [1,2] and Valentina Orlando [1,2]

Affiliations
[1] Department of Pharmacy, University of Naples Federico II, 80131 Naples, Italy; marianna.serino@unina.it (M.S.); milana.krstin@unina.it (M.K.); enrica.menditto@unina.it (E.M.); valentina.orlando@unina.it (V.O.)
[2] Center of Pharmacoeconomics and Drug Utilization Research (CIRFF), University of Naples Federico II, 80131 Naples, Italy
* Correspondence: sara.mucherino@unina.it

The supplementary material contains:

Table S1. Overview of ATMP Types and Their Regulatory Definitions

Table S2. Summary of all EMA authorized ATMPs with orphan designation.

Table S3. Search Strategy.

Table S4. Search Syntaxes.

Table S5. Characteristics of included studies.

Table S6. Key features of economic methods for the overall included studies.

Table S7. Assessment of risk of bias and quality of evidence.

Table S8. Quality appraisal of individual included studies according to Checklist.

Table S1: Overview of ATMP Types and Their Regulatory Definitions [2]

ATMP classification	Definition	Indications
**GTMP**	Biological medicinal product which has an active substance that contains: a recombinant nucleic acid used in or administered to human beings to regulate, repair, replace, add to, or delete a genetic sequence.	Therapeutic, prophylactic, or diagnostic effect relates directly to the recombinant nucleic acid sequence it contains, or to the product of genetic expression of this sequence.
**sCTMP**	Biological medicinal product that contains or consists of cells or tissues that have been substantially manipulated (altering biological characteristics, physiological functions, or structure), or not used for the same function in the donor and recipient.	Treating, preventing or diagnosing a disease through the pharmacological, immunological, or metabolic action of its cells or tissues.
**TEP**	A product that contains or consists of engineered cells or tissues.	Regenerating, repairing, or replacing a human tissue.
**cATMP**	A product that must incorporate, as an integral part of the product, one or more medical devices	Treting, repairing, or regenerating human tissues or functions where the biological (cell/tissue) component works in combination with a medical device, and the biological part has the primary therapeutic effect.

Abbreviations: GTMP: Gene therapy medicinal product; sCTM: Somatic cell therapy medicinal product; TEP: Tissue engineered product; cATMP: ‘Combined advanced therapy medicinal product

Note: The table presents the classification of Advanced Therapy Medicinal Products (ATMPs) according to Regulation (EC) No 1394/2007 and Directive 2001/83/EC.

Table S2. Summary of all EMA-authorized ATMPs with orphan designation. [5]

INN-International Nonproprietary Name	Trade Name	Type of Gene Therapy	Approved Indications	Authorization Date	Orphan designation
Alipogene tiparvovec	Glybera®*	GTMP	Treatment of diagnosed Familial lipoprotein lipase deficiency (LPLD)	25/10/2012	Yes
Talimogene laherparepvec	Imlygic®	GTMP	Treatment for metastatic melanoma	16/12/2015	No
autologous CD34+ enriched cell fraction that contains CD34+ cells transduced with retroviral vector that encodes for the human ADA cDNA sequence	Strimvelis®	GTMP	Treatment of the adenosine deaminase deficiency (ADA-SCID)	26/05/2016	Yes
Axicabtagene ciloleucel	Yescarta®	GTMP	Treatment of high-grade B-cell lymphoma (HGBL), diffuse large B-cell lymphoma (DLBCL), primary mediastinal large B-cell lymphoma (PMBCL), follicular lymphoma (FL)	23/08/2018	Yes
Exagamglogene autotemcel	Casgevy®	GTMP	Treatment of sickle cell disease and transfusion-dependent beta-thalassemia	9/02/2024	Yes
Fidanacogene elaparvovec	Durveqtix®	GTMP	Treatment of severe or moderately severe hemophilia B	24/07/2024	No
Voretigene neparvovec	Luxturna®	GTMP	Treatment of loss of vision due to inherited retinal dystrophy	22/11/2018	Yes
Tisagenlecleucel	Kymriah®	GTMP	Treatment of B-cell acute lymphoblastic leukemia (ALL), relapsed or refractory diffuse large B-cell lymphoma (DLBCL), relapsed or refractory follicular lymphoma (FL)	23/08/2018	Yes
Betibeglogene autotemcel	Zynteglo®*	GTMP	Treatment of beta thalassaemia	29/05/2019	Yes
Onasemnogene abeparvovec	Zolgensma®	GTMP	Treatment of spinal muscular atrophy	18/05/2020	Yes
Autologous CD34+ cells encoding ARSA gene	Libmeldy®	GTMP	Treatment of metachromatic leukodystrophy (MLD)	17/12/2020	Yes

INN-International Nonproprietary Name	Trade Name	Type of Gene Therapy	Approved Indications	Authorization Date	Orphan designation
Brexcabtagene autoleucel	Tecartus®	GTMP	Treatment of adults with mantle cell lymphoma and B-cell precursor acute lymphoblastic leukemia (ALL)	14/12/2020	Yes
Elivaldogene autotemcel	Skysona®*	GTMP	Treatment of metachromatic leukodystrophy (MLD)	16/07/2021	Yes
Idecabtagene vicleucel	Abecma®	GTMP	Treatment of relapsed and refractory multiple myeloma	18/08/2021	Yes
Ciltacabtagene autoleucel	Carvykti®	GTMP	Treatment of refractory and relapsed multiple myeloma	25/05/2022	Yes
Eladocagene exuparvovec	Upstaza®	GTMP	Treatment of aromatic L-amino acid decarboxylase (AADC) deficiency	18/07/2022	Yes
Lisocabtagene maraleucel	Breyanzi®	GTMP	Treatment of diffuse large B-cell lymphoma (DLBCL), high-grade B-cell lymphoma (HGBCL), primary mediastinal large B-cell lymphoma (PMBCL), and follicular lymphoma grade 3B (FL3B)	4/04/2022	No
Valoctocogene roxaparvovec	Roctavian®	GTMP	Treatment of severe hemophilia A	24/08/2022	Yes
Etranacogene dezaparvovec	Hemgenix®	GTMP	Treatment of severe and moderately severe hemophilia B	20/02/2023	Yes
Tabelecleucel	Ebvallo®	sCTMP	Treatment of Epstein-Barr virus-positive post-transplant lymphoproliferative disease (EBV+ PTLD)	16/12/2022	Yes
allogeneic T cells genetically modified with a retroviral vector encoding for a truncated form of the human low-affinity nerve growth factor receptor (ΔLNGFR) and the herpes simplex I virus thymidine kinase (HSV-TK Mut2)	Zalmoxis®*	sCTMP	Treatment in adults who have received a hematopoietic stem cell transplant from a partially matched donor	18/08/2016	Yes

INN-International Nonproprietary Name	Trade Name	Type of Gene Therapy	Approved Indications	Authorization Date	Orphan designation
Sipuleucel-T	Provenge®*	sCTMP	Treatment for adult men with minimally symptomatic metastatic castration-resistant prostate cancer	06/09/2013	No
Darvadstrocel	Alofisel®	sCTMP	Treatment of complex perianal fistulas in adult patients with non-active/mildly active Crohn's disease	23/03/2018	Yes
characterized viable autologous cartilage cells expanded ex vivo expressing specific marker proteins Medicine Human	Chondrocelect®*	TEP	Treatment of joint cartilage defects	5/10/2009	No
Matrix applied characterised autologous cultured chondrocytes	MACI®*	TEP, combined ATMP	Repair of symptomatic cartilage damage of the adult knee.	27/06/2013	No
Ex vivo expanded autologous human corneal epithelial cells containing stem cells	Holoclar®	TEP	Treatment for the eye to replace damaged cells on the surface of the cornea	17/02/2015	Yes
Spheroids of human autologous matrix-associated chondrocytes	Spherox®	TEP	Repair of symptomatic articular cartilage defects of the femoral condyle and the patella of the knee	10/07/2017	No

**Withdrawn or not renewed authorization*

Abbreviations: GTMP: Gene therapy medicinal product; sCTM: Somatic cell therapy medicinal product; TEP: Tissue engineered product.

Table S3. Search Strategy

Query	Keywords	Field	Database			
			PubMed	Embase	ProQuest	Web of Science
#1	Advanced therapy medicinal products OR ATMPs OR Holoclar OR Strimvelis OR Alofisel OR Yescarta OR Kymriah OR Luxturna OR Zolgensma OR Libmeldy OR Tecartus OR Abecma OR Carvykti OR Upstaza OR Roctavian OR Ebvallo OR Hemgenix OR Casgevy OR Gene therapy OR CAR-T OR Cell therapy OR Tissue Engineering OR Cell- and Tissue-Based Therapy	MeSH terms/Emtree	1,613	1,377,684	1.002.828	446,265
#2	Ex vivo expanded autologous human corneal epithelial OR cells containing stem cells OR autologous CD34+ enriched cell fraction OR Darvadstrocel OR Axicabtagene ciloleucel OR Tisagenlecleucel OR Voretigene neparvovec OR Onasemnogene abeparvovec OR Atidarsagene autotemcel OR Brexucabtagene autoleucel OR Idecabtagene vicleucel OR Ciltacabtagene autoleucel OR Eladocagene exuparvovec OR Valoctocogene roxaparvovec OR Tabelecleucel OR Etranacogene dezaparvovec OR Exagamglogene autotemcel	MeSH terms/Emtree OR Title and Abstract	1,530	1,377,684	315.642	23,568
#3	Economic evaluation OR Economic burden OR Cost-effectiveness analysis OR cost-benefit OR cost utility OR cost analysis OR Quality of life OR quality-adjusted life year OR QALY OR ICER OR incremental cost-effectiveness ratio OR ICUR OR incremental cost-utility ratio OR cost	MeSH terms/Emtree	1,241	1,075,735	2.747.298	922,572
**#4**	**#1 AND #2 AND #3**		**380**	**337**	**328**	**491**

Table S4. Search Syntaxes

Database	Query executed
**Pubmed**	((("cost benefit analysis"[MeSH Terms] OR ("cost benefit"[All Fields] AND "analysis"[All Fields]) OR "cost benefit analysis"[All Fields] OR ("economic"[All Fields] AND "evaluation"[All Fields]) OR "economic evaluation"[All Fields] OR ("financial stress"[MeSH Terms] OR ("financial"[All Fields] AND "stress"[All Fields]) OR "financial stress"[All Fields] OR ("economic"[All Fields] AND "burden"[All Fields]) OR "economic burden"[All Fields]) OR ("cost effectiveness analysis"[MeSH Terms] OR ("cost effectiveness"[All Fields] AND "analysis"[All Fields]) OR "cost effectiveness analysis"[All Fields] OR ("cost"[All Fields] AND "effectiveness"[All Fields] AND "analysis"[All Fields]) OR "cost effectiveness analysis"[All Fields]) OR ("cost benefit analysis"[MeSH Terms] OR ("cost benefit"[All Fields] AND "analysis"[All Fields]) OR "cost benefit analysis"[All Fields] OR ("cost"[All Fields] AND "benefit"[All Fields]) OR "cost benefit"[All Fields]) OR (("economics"[MeSH Subheading] OR "economics"[All Fields] OR "cost"[All Fields] OR "costs and cost analysis"[MeSH Terms] OR ("costs"[All Fields] AND "cost"[All Fields] AND "analysis"[All Fields]) OR "costs and cost analysis"[All Fields]) AND ("statistics and numerical data"[MeSH Subheading] OR ("statistics"[All Fields] AND "numerical"[All Fields] AND "data"[All Fields]) OR "statistics and numerical data"[All Fields] OR "utilization"[All Fields] OR "utilisation"[All Fields] OR "utilisations"[All Fields] OR "utilise"[All Fields] OR "utilised"[All Fields] OR "utilises"[All Fields] OR "utilising"[All Fields] OR "utilities"[All Fields] OR "utility"[All Fields] OR "utilizations"[All Fields] OR "utilize"[All Fields] OR "utilized"[All Fields] OR "utilizer"[All Fields] OR "utilizers"[All Fields] OR "utilizes"[All Fields] OR "utilizing"[All Fields])) OR ("costs and cost analysis"[MeSH Terms] OR ("costs"[All Fields] AND "cost"[All Fields] AND "analysis"[All Fields]) OR "costs and cost analysis"[All Fields] OR ("cost"[All Fields] AND "analysis"[All Fields]) OR "cost analysis"[All Fields]) OR ("quality of life"[MeSH Terms] OR ("quality"[All Fields] AND "life"[All Fields]) OR "quality of life"[All Fields]) OR ("quality adjusted life years"[MeSH Terms] OR ("quality adjusted"[All Fields] AND "life"[All Fields] AND "years"[All Fields]) OR "quality adjusted life years"[All Fields] OR ("quality"[All Fields] AND "adjusted"[All Fields] AND "life"[All Fields] AND "year"[All Fields]) OR "quality adjusted life year"[All Fields]) OR ("quality adjusted life years"[MeSH Terms] OR ("quality adjusted"[All Fields] AND "life"[All Fields] AND "years"[All Fields]) OR "quality adjusted life years"[All Fields] OR "qaly"[All Fields]) OR "ICER"[All Fields] OR (("incremental"[All Fields] OR "incrementally"[All Fields]) AND ("cost effectiveness analysis"[MeSH Terms] OR ("cost effectiveness"[All Fields] AND "analysis"[All Fields]) OR "cost effectiveness analysis"[All Fields] OR ("cost"[All Fields] AND "effectiveness"[All Fields] AND "ratio"[All Fields]) OR "cost effectiveness ratio"[All Fields])) OR "ICUR"[All Fields] OR (("incremental"[All Fields] OR "incrementally"[All Fields]) AND "cost-utility"[All Fields] AND ("ratio"[All Fields] OR "ratio s"[All Fields] OR "ratioes"[All Fields] OR "ratios"[All Fields])) OR "costs and cost analysis"[MeSH Terms]) AND ("loattrfree full text"[Filter] AND ("case reports"[Publication Type] OR "classical article"[Publication Type] OR "preprint"[Publication Type]) AND "loattrfull text"[Filter])) OR ((("Ex"[All Fields] AND "vivo"[All Fields] AND ("expand"[All Fields] OR "expandable"[All Fields] OR "expanded"[All Fields] OR "expandible"[All Fields] OR "expanding"[All Fields] OR "expands"[All Fields]) AND ("autolog"[All Fields] OR "autologeous"[All Fields] OR "autologic"[All Fields] OR "autological"[All Fields] OR "autologous"[All Fields] OR "autologously"[All Fields]) AND ("human s"[All Fields] OR "humans"[MeSH Terms] OR "humans"[All Fields] OR "human"[All Fields]) AND ("cornea"[MeSH Terms] OR "cornea"[All Fields] OR "corneal"[All Fields]) AND ("epithelial"[All Fields] OR "epithelially"[All Fields] OR "epithelials"[All Fields])) OR (("cell s"[All Fields] OR "cells"[MeSH Terms] OR "cells"[All Fields]) AND ("contain"[All Fields] OR "contained"[All Fields] OR "container"[All Fields] OR "container s"[All Fields] OR "containers"[All Fields] OR "containing"[All Fields] OR "contains"[All Fields]) AND ("stem cells"[MeSH Terms] OR ("stem"[All Fields] AND "cells"[All Fields]) OR "stem cells"[All Fields])) OR (("autolog"[All Fields] OR "autologeous"[All Fields] OR "autologic"[All Fields] OR "autological"[All Fields] OR "autologous"[All Fields] OR "autologously"[All Fields]) AND "cd34"[All Fields] AND ("enrich"[All Fields] OR "enriched"[All Fields] OR "enriches"[All Fields] OR "enriching"[All Fields] OR "enrichment"[All Fields] OR "enrichments"[All Fields]) AND ("cells"[MeSH Terms] OR "cells"[All Fields] OR "cell"[All Fields]) AND "fractio"[All Fields]) OR "Darvadstrocel"[All Fields] OR ("axicabtagene ciloleucel"[Supplementary Concept] OR "axicabtagene ciloleucel"[All Fields]) OR ("Tisagenlecleucel"[Supplementary Concept] OR "Tisagenlecleucel"[All Fields]) OR ("Voretigene"[All Fields] AND "neparvovec"[All Fields]) OR ("zolgensma"[Supplementary Concept] OR "zolgensma"[All Fields] OR "onasemnogene abeparvovec"[All Fields]) OR ("Atidarsagene"[All Fields] AND "autotemcel"[All Fields]) OR

	("brexucabtagene autoleucel"[Supplementary Concept] OR "brexucabtagene autoleucel"[All Fields]) OR ("idecabtagene vicleucel"[Supplementary Concept] OR "idecabtagene vicleucel"[All Fields]) OR ("Ciltacabtagene"[All Fields] AND "autoleucel"[All Fields]) OR ("Eladocagene"[All Fields] AND "exuparvovec"[All Fields]) OR ("valoctocogene roxaparvovec"[Supplementary Concept] OR "valoctocogene roxaparvovec"[All Fields] OR "valoctocogene roxaparvovec"[All Fields]) OR "Tabelecleucel"[All Fields] OR ("Etranacogene"[All Fields] AND "dezaparvovec"[All Fields]) OR ((("Ex"[All Fields] AND "vivo"[All Fields] AND ("expand"[All Fields] OR "expandable"[All Fields] OR "expanded"[All Fields] OR "expandible"[All Fields] OR "expanding"[All Fields] OR "expands"[All Fields]) AND ("autolog"[All Fields] OR "autologeous"[All Fields] OR "autologic"[All Fields] OR "autological"[All Fields] OR "autologous"[All Fields] OR "autologously"[All Fields])) AND "human corneal epithelial"[Title/Abstract]) OR (("cell s"[All Fields] OR "cells"[MeSH Terms] OR "cells"[All Fields]) AND "containing stem cells"[Title/Abstract]) OR ((("autolog"[All Fields] OR "autologeous"[All Fields] OR "autologic"[All Fields] OR "autological"[All Fields] OR "autologous"[All Fields] OR "autologously"[All Fields]) AND "cd34"[All Fields] AND ("enrich"[All Fields] OR "enriched"[All Fields] OR "enriches"[All Fields] OR "enriching"[All Fields] OR "enrichment"[All Fields] OR "enrichments"[All Fields]) AND ("cells"[MeSH Terms] OR "cells"[All Fields] OR "cell"[All Fields])) AND "fractio"[Title/Abstract]) OR "Darvadstrocel"[Title/Abstract] OR "axicabtagene ciloleucel"[Title/Abstract] OR "Tisagenlecleucel"[Title/Abstract] OR "voretigene neparvovec"[Title/Abstract] OR "onasemnogene abeparvovec"[Title/Abstract] OR "atidarsagene autotemcel"[Title/Abstract] OR "brexucabtagene autoleucel"[Title/Abstract] OR "idecabtagene vicleucel"[Title/Abstract] OR "ciltacabtagene autoleucel"[Title/Abstract] OR "eladocagene exuparvovec"[Title/Abstract] OR "valoctocogene roxaparvovec"[Title/Abstract] OR "Tabelecleucel"[Title/Abstract] OR "etranacogene dezaparvovec"[Title/Abstract] OR "exagamglogene autotemcel"[Title/Abstract])) AND ("loattrfree full text"[Filter] AND ("case reports"[Publication Type] OR "classical article"[Publication Type] OR "preprint"[Publication Type]) AND "loattrfull text"[Filter])) OR (((("advance"[All Fields] OR "advanced"[All Fields] OR "advancement"[All Fields] OR "advancements"[All Fields] OR "advances"[All Fields] OR "advancing"[All Fields]) AND ("therapeutics"[MeSH Terms] OR "therapeutics"[All Fields] OR "therapies"[All Fields] OR "therapy"[MeSH Subheading] OR "therapy"[All Fields] OR "therapy s"[All Fields] OR "therapys"[All Fields]) AND ("pharmaceutical preparations"[MeSH Terms] OR ("pharmaceutical"[All Fields] AND "preparations"[All Fields]) OR "pharmaceutical preparations"[All Fields] OR ("medicinal"[All Fields] AND "products"[All Fields]) OR "medicinal products"[All Fields])) OR "ATMPs"[All Fields] OR "Holoclar"[All Fields] OR "Strimvelis"[All Fields] OR "Alofisel"[All Fields] OR ("axicabtagene ciloleucel"[Supplementary Concept] OR "axicabtagene ciloleucel"[All Fields] OR "yescarta"[All Fields]) OR ("Tisagenlecleucel"[Supplementary Concept] OR "Tisagenlecleucel"[All Fields] OR "kymriah"[All Fields]) OR ("luxturna"[All Fields] OR "voretigene neparvovec"[All Fields]) OR ("zolgensma"[Supplementary Concept] OR "zolgensma"[All Fields] OR "onasemnogene abeparvovec"[All Fields] OR "zolgensma"[All Fields]) OR "Libmeldy"[All Fields] OR ("brexucabtagene autoleucel"[Supplementary Concept] OR "brexucabtagene autoleucel"[All Fields] OR "tecartus"[All Fields]) OR ("abecma"[All Fields] OR "idecabtagene vicleucel"[Supplementary Concept] OR "idecabtagene vicleucel"[All Fields]) OR "Carvykti"[All Fields] OR "Upstaza"[All Fields] OR "Roctavian"[All Fields] OR "Ebvallo"[All Fields] OR "Hemgenix"[All Fields] OR "Casgevy"[All Fields] OR ("genetic therapy"[MeSH Terms] OR ("genetic"[All Fields] AND "therapy"[All Fields]) OR "genetic therapy"[All Fields] OR ("gene"[All Fields] AND "therapy"[All Fields]) OR "gene therapy"[All Fields]) OR "CAR-T"[All Fields] OR ("cell and tissue based therapy"[MeSH Terms] OR ("cell"[All Fields] AND "tissue based"[All Fields] AND "therapy"[All Fields]) OR "cell and tissue based therapy"[All Fields] OR ("cell"[All Fields] AND "therapy"[All Fields]) OR "cell therapy"[All Fields]) OR ("tissue engineering"[MeSH Terms] OR ("tissue"[All Fields] AND "engineering"[All Fields]) OR "tissue engineering"[All Fields]) OR "cell and tissue based therapy"[MeSH Terms]) AND ("loattrfree full text"[Filter] AND ("case reports"[Publication Type] OR "classical article"[Publication Type] OR "preprint"[Publication Type]) AND "loattrfull text"[Filter]))) AND ((ffrft[Filter]) AND (casereports[Filter] OR classicalarticle[Filter] OR preprint[Filter]) AND (fft[Filter]))
**Embase**	('ex vivo expanded autologous human corneal epithelial' OR 'cells containing stem cells' OR 'autologous cd34+ enriched cell fractio' OR 'darvadstrocel' OR 'axicabtagene ciloleucel' OR 'tisagenlecleucel' OR 'voretigene neparvovec' OR 'onasemnogene abeparvovec' OR 'atidarsagene autotemcel' OR 'brexucabtagene autoleucel' OR 'idecabtagene vicleucel' OR 'ciltacabtagene autoleucel' OR 'eladocagene exuparvovec' OR 'valoctocogene roxaparvovec' OR 'tabelecleucel' OR 'etranacogene dezaparvovec' OR 'exagamglogene autotemcel') AND ('advanced therapy medicinal

	products' OR 'atmps' OR 'holoclar'/exp OR 'holoclar' OR 'strimvelis'/exp OR 'strimvelis' OR 'alofisel'/exp OR 'alofisel' OR 'yescarta'/exp OR 'yescarta' OR 'kymriah'/exp OR 'kymriah' OR 'luxturna'/exp OR 'luxturna' OR 'zolgensma'/exp OR 'zolgensma' OR 'libmeldy'/exp OR 'libmeldy' OR 'tecartus'/exp OR 'tecartus' OR 'abecma'/exp OR 'abecma' OR 'carvykti'/exp OR 'carvykti' OR 'upstaza'/exp OR 'upstaza' OR 'roctavian'/exp OR 'roctavian' OR 'ebvallo'/exp OR 'ebvallo' OR 'hemgenix'/exp OR 'hemgenix' OR 'casgevy'/exp OR 'casgevy' OR 'gene therapy'/exp OR 'gene therapy' OR 'car-t' OR 'cell therapy'/exp OR 'cell therapy' OR 'tissue engineering'/exp OR 'tissue engineering' OR 'cell- and tissue-based therapy'/exp OR 'cell- and tissue-based therapy') AND ('economic evaluation' OR 'economic burden' OR 'cost-effectiveness analysis' OR 'cost-benefit' OR 'cost utility' OR 'cost analysis' OR 'quality of life' OR 'quality-adjusted life year' OR 'qaly' OR 'icer' OR 'incremental cost-effectiveness ratio' OR 'icur' OR 'incremental cost-utility ratio' OR 'cost') AND ('article'/it OR 'article in press'/it OR 'preprint'/it)
**ProQuest**	Advanced therapy medicinal products OR ATMPs OR Holoclar OR Strimvelis OR Alofisel OR Yescarta OR Kymriah OR Luxturna OR Zolgensma OR Libmeldy OR Tecartus OR Abecma OR Carvykti OR Upstaza OR Roctavian OR Ebvallo OR Hemgenix OR Casgevy OR Gene therapy OR CAR-T OR Cell therapy OR Tissue Engineering OR Cell- AND Tissue-Based Therapy) AND (Ex vivo expanded autologous human corneal epithelial OR cells containing stem cells OR autologous CD34+ enriched cell fractio OR Darvadstrocel OR Axicabtagene ciloleucel OR Tisagenlecleucel OR Voretigene neparvovec OR Onasemnogene abeparvovec OR Atidarsagene autotemcel OR Brexucabtagene autoleucel OR Idecabtagene vicleucel OR Ciltacabtagene autoleucel OR Eladocagene exuparvovec OR Valoctocogene roxaparvovec OR Tabelecleucel OR Etranacogene dezaparvovec OR Exagamglogene autotemcel) AND (Economic evaluation OR Economic burden OR Cost-effectiveness analysis OR cost-benefit OR cost utility OR cost analysis OR Quality of life OR quality-adjusted life year OR QALY OR ICER OR incremental cost-effectiveness ratio OR ICUR OR incremental cost-utility ratio OR cost)
**Web of Science**	((ALL=(Economic evaluation OR Cost-effectiveness analysis OR cost-benefit OR cost utility OR cost analysis OR Quality of life OR quality-adjusted life year OR QALY OR ICER OR incremental cost-effectiveness ratio OR ICUR OR incremental cost-utility ratio OR cost)) AND ALL=(Ex vivo expanded autologous human corneal epithelial OR cells containing stem cells OR autologous CD34+ enriched cell fractio OR Darvadstrocel OR Axicabtagene ciloleucel OR Tisagenlecleucel OR Voretigene neparvovec OR Onasemnogene abeparvovec OR Atidarsagene autotemcel OR Brexucabtagene autoleucel OR Idecabtagene vicleucel OR Ciltacabtagene autoleucel OR Eladocagene exuparvovec OR Valoctocogene roxaparvovec OR Tabelecleucel OR Etranacogene dezaparvovec OR Exagamglogene autotemcel)) AND ALL=(Advanced therapy medicinal products OR ATMPs OR Holoclar OR Strimvelis OR Alofisel OR Yescarta OR Kymriah OR Luxturna OR Zolgensma OR Libmeldy OR Tecartus OR Abecma OR Carvykti OR Upstaza OR Roctavian OR Ebvallo OR Hemgenix OR Casgevy OR Gene therapy OR CAR-T OR Cell therapy)

Table S5. Characteristics of included studies.

References; Year; Country	ATMP	Diagnosis	Comparator(s)	Original ICER/ICUR per QALY	ICER/ICUR per QALY €2023 Adjustment	Cost-effective
**Blood cancer and hemophilia B**						
Joshua A. Roth et al. 2018, US [19]	Axicabtagene ciloleucel	DLBCL	Chemotherapy	$58,146/ QALY	€50,182/QALY	Yes
Rongzhe Liu et al. 2021; US [20]	Axicabtagene ciloleucel	DLBCL	Tisagenlecleucel	$–609/ QALY	€-503 /QALY	Yes
Melanie D. Whittington et al. 2019, US [21]	Axicabtagene ciloleucel	DLBCL	Chemotherapy	-Standard Parametric: $ 230 900/QALY -Flexible Parametric: $ 111 200/ QALY -Mixture Cure: $100 900/QALY -Mixture Cure: $82 400/QALY -Flexible Parametric Mixture: $148 800/ QALY	-Standard Parametric: €199,274/QALY -Flexible Parametric: €95,969/QALY -Mixture Cure: €87,079/QALY -Mixture Cure: €71,113/QALY -Flexible Parametric Mixture: €128,419/QALY	NR
Olalekan O. Oluwole et al. 2022 ; US [22]	Axicabtagene ciloleucel	DLBCL	Lisocabtagene maraleucel	$7,843/QALY	€6,134/QALY	Yes
Jee H. Choe et al. 2022; US [23]	-Axicabtagene ciloleucel, -Tisagenlecleucel	DLBCL	SoC	-Axicabtagene ciloleucel vs SoC (second line): $99 101/ QALY	-Axicabtagene ciloleucel vs SoC (second line): €77,509 / QALY	Yes
				-Tisagenlecleucel vs SoC (second line): $1,890,150/ QALY	-Tisagenlecleucel vs SoC (second line): €-1,478,300/ QALY	No
				-Tisagenlecleucel vs SoC (third line): $ 126,593/QALY	-Tisagenlecleucel vs SoC (third line): €99,011/QALY	Yes
	Axicabtagene ciloleucel	DLBCL	-Tisagenlecleucel	-Tisagenlecleucel : $24,506/QALY	-Tisagenlecleucel : 20,027/QALY	Yes

Alice Kate Cummings Joyner et al. 2022; US [24]			-Lisocabtagene maraleucel	-Lisocabtagene maraleucel : $8946/QALY	-Lisocabtagene maraleucel : €7,311/QALY	
Christopher Hillis et al. 2022; Canada[25]	Axicabtagene ciloleucel	DLBCL	BSC	C$132,747/QALY	€67,129/QALY	Yes
Swetha Kambhampati et al. 2022; US[26]	Axicabtagene ciloleucel	DLBCL	SoC	$93,547/QALY	€73,165/QALY	Yes
Miguel-Angel Perales et al. 2022 ; US[27]	Axicabtagene ciloleucel	DLBCL	SoC	$66,381/QALY	€51,918/QALY	Yes
Na li et al. 2022; China [28]	Axicabtagene ciloleucel	DLBCL	Chemotherapy	$67,250/QALY	€54,961/QALY	No
Mariana Baston-Oreiro et al.2022; Spain[29]	Axicabtagene ciloleucel	DLBCL	Tisagenlecleucel	€13,049/QALY	€16,220/QALY	Yes
Weijia Wu et al. 2023 ; China [30]	-Axicabtagene ciloleucel<br>-Tisagenlecleucel<br>-Lisocabtagene maraleucel	DLBCL	SoC	-Axicabtagene ciloleucel 1st line vs SoC: CNY 2,125,311/QALY<br>-Axicabtagene ciloleucel 2nd line vs SoC: CNY 363,977/QALY<br>-Axicabtagene ciloleucel 3rd line vs SoC: CNY 346,009/QALY<br>-Tisagenlecleucel 2nd line vs SoC: CNY 32,066,781/QALY<br>-Tisagenlecleucel 3rd line vs SoC: CNY 654,344/QALY<br>-Lisocabtagene maraleuce 2nd line vs SoC: l: CNY 347,746/QALY<br>-Lisocabtagene maraleucel 3rd line vs SoC:CNY 436,858/QALY	-Axicabtagene ciloleucel 1st line vs SoC: €429,577/QALY<br>-Axicabtagene ciloleucel 2nd line vs SoC: €73,568/QALY<br>-Axicabtagene ciloleucel 3rd line vs SoC: €68,456/QALY<br>-Tisagenlecleucel 2nd line vs SoC: €6,481,481/QALY<br>-Tisagenlecleucel 3rd line vs SoC: €129,459/QALY<br>-Lisocabtagene maraleuce 2nd line vs SoC: l: €70,28 7/QALY<br>-Lisocabtagene maraleucel 3rd line vs SoC: €357,025/QALY	No
Anne sofie Ledgaard Loftager et al. 2023 ; Sweden [31]	Axicabtagene ciloleucel	DLBCL	SoC	SEK 534,704/QALY	€45,408/QALY	Yes
Na li et al. 2023; US and China [32]	Axicabtagene ciloleucel	DLBCL	SoC in China	- SoC China: $45,726 /QALY	-SoC China: €35,76/QALY	No

			SoC in the US	- SoC US: $142,326 /QALY	-SoC US: €111,318/QALY	Yes
Olalekan O. Oluwole et al. 2024 ; US [33]	Axicabtagene ciloleucel	DLBCL	SoC	$98,040/ QALY	€69,118/QALY	Yes
Alejandro Martín Garcia Sancho et al. 2024 ; Spain [34]	Axicabtagene ciloleucel	DLBCL	SoC	€47,309/QALY	€55,005/QALY	Yes
Olalekan O. Oluwole et al. 2024; US [35]	Axicabtagene ciloleucel	DLBCL	Tisagenlecleucel	$19,994/ QALY	€14,614 /QALY	Yes
Markqayne Ray et al. 2024 ; France [36]	Axicabtagene ciloleucel	DLBCL	Tisagenlecleucel	€15,520/QALY	€14,776/QALY	Yes
Saaya Tsutsue et al. 2024 ; Japan [37]	Axicabtagene ciloleucel	DLBCL	-Tisagenlecleucel<br>-Lisocabtagene maraleucel	-Tisagenlecleucel : €--368/QALY<br>-Lisocabtagene maraleucel :<br>€-108/QALY	-Tisagenlecleucel : €-260/QALY<br>-Lisocabtagene maraleucel :<br>€-76/QALY	Yes
Saaya Tsutsue et al. 2024 ; Japan [38]	Axicabtagene ciloleucel	DLBCL	SoC	$23,590/ QALY	€16,631/QALY	Yes
Kunal C. Potnis et al. 2023 ; US [39]	Axicabtagene ciloleucel	FL	SoC	$182,127/QALY	€142,446 /QALY	No
Olalekan O. Oluwole et al. 2024 ; US [40]	Axicabtagene ciloleucel	FL	Mosunetuzumab	$108,307/QALY	€79,164/QALY	Yes
Olalekan O. Oluwole et al.2024; US [41]	Axicabtagene ciloleucel	FL	SoC	$88-,300/ QALY	€64,540/QALY	Yes
Melanie D. Whittington et al. 2018; US [43]	Tisagenlecleucel	ALL	Clofarabine	$45,871/QALY	€39,588/QALY	Yes
Reith R. Sarkar et al. 2019 ; US [44]	Tisagenlecleucel	ALL	SoC	$64 600/QALY	€55,752/QALY	Yes
Frederick W. Thielen et al. 2020 ;Netherlands [45]	Tisagenlecleucel	ALL	-Clofarabine mono<br>-Clofarabine combination<br>-Blinatumomab	-Clofarabine mono: €36,378/ QALY<br>-Clofarabine combination €37,531/ QALY<br>-Blinatumomab: €31,682/QALY	-Clofarabine mono: €36,828/QALY<br>-Clofarabine combination therapy: €37,996/QALY<br>-Blinatumomab: €32,074/QALY	Yes
Jill Furzer et al. 2020 ; Canada [78]	Tisagenlecleucel	ALL	SoC	-0.1% cure rate: C$ 281,000/QALY<br>-0.2% cure rate: C$ 141,000/QALY<br>-0.4% cure rate: C$71 000/QALY	-0.1% cure rate: €195,958/QALY<br>-0.2% cure rate: €98,327/QALY<br>-0.4% cure rate: €49,512/QALY	No

Josep Maria Ribera Santasusana et al. 2020; Spain [46]	Tisagenlecleucel	ALL	Chemotherapy	€28,818/ QALY	€36,753/QALY	Yes
Shiho Wakase et al. 2021; Japan [47]	Tisagenlecleucel	ALL	-Blinatumomab<br>-Clofarabine	-Blinatumomab: ¥2,035,071/ QALY<br>-Clofarabine: ¥2,644,702/QALY	-Blinatumomab: €16 351/QALY<br>-Clofarabine: €21,249/QALY	Yes
Maziar Moradi-laken et al. 2021 ; Switzerland [48]	Tisagenlecleucel	ALL<br>DLBCL	-Chemotherapy<br>-Clofarabine<br>-Blinatumomab<br>-SoC	-Chemotherapy: CHF 31,961/QALY<br>-Clofarabine: CHF 34,530/ QALY<br>-Blinatumomab: CHF 36,419/QALY<br>-SoC: CHF 113,179/QALY	-Chemotherapy : €19,857 /QALY<br>-Clofarabine : €21,453 /QALY<br>-Blinatumomab : €22,627/QALY<br>-SoC: €70,318/QALY	Yes
Xiao Jun Wang et al. 2021 ; Singapore [49]	Tisagenlecleucel	ALL	Chemotherapy	S$-3,049/QALY	€3,141/QALY	Yes
Niamh Carey et al. 2022; Ireland [63]	Tisagenlecleucel	ALL	Blinatumomab	€73,086/ QALY	€71,370/QALY	No
Amy Gye et al. 2022; Australia [72]	Tisagenlecleucel	ALL	SoC	$90,129/QALY	€65,877*/QALY	NR
Amy Gye et al. 2024; Australia [67]	Tisagenlecleucel	ALL	SoC	$96,074/QALY	€67,732/QALY	NR
Amy Gye et al. 2024; Australia [65]	Tisagenlecleucel	ALL	SoC	-State-transition model: $99,625/QALY<br>-Partitioned survival model: $99,038/QALY<br>-Discrete event simulation: $96,074/QALY	-State-transition model: €49,384/QALY<br>-Partitioned survival model: €49,081/QALY<br>-Discrete event simulation: €47,560/QALY	NR
Boon Piang Cher et al. 2020; Singapore [71]	Tisagenlecleucel	DLBCL	SoC	$508,530/QALY	€428,577/QALY	No
Shiho Wakase et al. 2021 ; Japan [79]	Tisagenlecleucel	DLBCL	Chemotherapy	¥5,476,496/QALY	€44,279 / QALY	Yes
Cynthia Z. Qi et al. 2021 ; US [70]	Tisagenlecleucel	DLBCL	SoC	$78,652/QALY	€64,279/QALY	Yes
	Tisagenlecleucel	DLBCL	-SoC	-SoC: S$45,840/QALY	-SoC: €47,222/QALY	Yes

Xiao Jun Wang et al. 2022; Singapore [69]			-Blinatumomab	-Blinatumomab: S$51,978/QALY	-Blinatumomab: €53,545 / QALY	
Niamh Carey et al. 2023; Ireland [64]	Tisagenlecleucel	DLBCL	Chemotherapy	€119,509/QALY	€116,703/QALY	No
Lisa Masucci et al. 2024; Canada [61]	Tisagenlecleucel	DLBCL	Chemotherapy	C$202,991/QALY	€128,079/QALY	No
Weijia Wu et al. 2023; China [55]	-Idecabtagene vicleucel<br>-Ciltacabtagene autoleucel	MM	Chemotherapy	-Idecabtagene vicleucel vs Chemotherapy : $118,229/QALY<br>-Ciltacabtagene autoleucel vs Chemotherapy : $36,195/QALY	-Idecabtagene vicleucel vs Chemotherapy : €92,467/QALY<br>-Ciltacabtagene autoleucel vs Chemotherapy : €28,309/QALY	No<br>Yes
Kandice A. Kapinos et al. 2023 ; US [56]	-Idecabtagene vicleucel<br>-Ciltacabtagene autoleucel	MM	Belamaf	-Idecabtagene vicleucel vs Belamaf : $ 182,535/QALY<br>-Ciltacabtagene autoleucel vs Belamaf : $123,618/QALY	-Idecabtagene vicleucel vs Belamaf : €149,178/QALY<br>-Ciltacabtagene autoleucel vs Belamaf : €101, 027/QALY	NR<br>Yes
Korinna Karampampa et al. 2023; Canada and France [74]	Idecabtagene vicleucel	MM	-SoC in Canada<br>-SoC in France	-SoC in Canada: C$255,245/QALY<br>-SoC in France: €154,593/QALY	-SoC in Canada: €161,050/QALY<br>-SoC in France: €151,507/QALY	NR
Chihiro Yamamoto et al. 2024-; Japan, US [57]	-Idecabtagene vicleucel<br>-Ciltacabtagene autoleucel	MM	-Chemotherapy in Japan<br>-Chemotherapy in US	-Idecabtagene vicleucel vs Chemotherapy in Japan: ¥ 20,388,711/QALY**<br>-Idecabtagene vicleucel vs Chemotherapy in US: 261,678/QALY**<br>-Ciltacabtagene autoleucel vs Chemotherapy Japan: ¥5,778,608/QALY**<br>-Ciltacabtagene autoleucel vs Chemotherapy US: $85,553/QALY**	-Idecabtagene vicleucel vs Chemotherapy in Japan: €156,802 /QALY**<br>-Idecabtagene vicleucel vs Chemotherapy in US: €144,409/QALY**<br>-Ciltacabtagene autoleucel vs Chemotherapy Japan: €44,354/QALY**<br>-Ciltacabtagene autoleucel vs Chemotherapy US: €60,273 /QALY**	No<br>No<br>Yes<br>Yes
Claire L. Simons et al 2021 ; US [54]	Brexucabtagene autoleucel	MCL	SoC	$31,985/QALY	€26,140/QALY	Yes

Graeme Ball et al. 2022 ; Canada [50]	Brexucabtagene autoleucel	MCL	BSC	C$ 88,503/QALY	€55,841/QALY	Yes
Svenja Petersohn et al. 2022; UK [51]	Brexucabtagene autoleucel	MCL	SoC	£67,713/QALY	€76,656/QALY	Yes
M. Marchetti et al. 2023; Italy [52]	Brexucabtagene autoleucel	MCL	SoC	€64,798/ QALY	€67,720/QALY	Yes
Bijal D. Shah et al. 2022 ; US [53]	Brexucabtagene autoleucel	ALL	-Blinatumomab,<br>-Inotuzumab ozogamicin<br>-Chemotherapy	-Blinatumomab, : $20,843/QALY<br>-Inotuzumab ozogamicin : $77,271/QALY<br>-Chemotherapy : $93,768/QALY	-Blinatumomab,: €16,301/QALY<br>-Inotuzumab ozogamicin : €60,435/QALY<br>-Chemotherapy : €73,338/QALY	Yes
Christopher Parker et al. 2023; US [75]	Lisocabtagene maraleucel	DLBCL	-Axicabtagene ciloleucel<br>-Tisagenlecleucel	-Axicabtagene ciloleucel:<br>$ - 37,490,000/QALY<br>-Tisagenlecleucel: $33,618/QALY	-Axicabtagene ciloleucel:<br>-Tisagenlecleucel: €27,474/QALY	Yes
Niklaus Meier et al. 2024 ; Germany [42]	Etranacogene dezaparvovec	Hemophilia B	SoC	€-2,036,441/QALY	€-1,937,936/QALY	Yes
**Spinal Muscular Atrophy Type I**						
Daniel C. Malone et al. 2019 ; US [62]	Onasemnogene abeparvocec	SMA	Nusinersen	$31,379/QALY	€25,979/ QALY	Yes
Thomas F. Broekhoff et al. 2021; Netherlands [68]	Onasemnogene abeparvocec	SMA	-BSC<br>-Nusinersen	-BSC: €138,875/QALY<br>-Nusinersen: €53,447/QALY	-BSC: €143, 304/QALY<br>-Nusinersen: €55, 152 /QALY	No<br>Yes
Rebecca Dean et al. 2021; US [77]	Onasemnogene abeparvocec	SMA	-Nusinersen<br>-BSC	-Nusinersen:<br>-$64,121/QALY<br>-BSC:<br>$ 294,889/QALY	-Nusinersen: €-53,073 /QALY<br>-BSC: €133,832 /QALY	Yes<br>No
Tianjiao Wang et al. 2022, Australia [76]	Onasemnogene abeparvocec	SMA	-SoC<br>-Nusinersen	-SoC: A$1,808,471/QALY<br>-Nusinersen: A$1,238,288/QALY	-SoC: €1,020,693/QALY<br>-Nusinersen: €698,884/QALY	No
**Retinal diseases with RP E65-mutation**						
Marita Zimmermann et al. 2019, US [66]	Voretigene neparvovec	RPE65-IRD	SoC	$643,813/QALY	€555,631/QALY	No
Scott Johnson et al. 2019; US [58]	Voretigene neparvovec	RPE65-IRD	SoC	$−59,458/QALY	€-50,211/QALY	Yes

Matthias Fritz Uhrmann et al. 2020; Germany [59]	Voretigene neparvovec	RPE65-IRD	SoC	€156,853/ QALY	€161,870/QALY	Yes
Daniel Viriato et al. 2020 ; UK [60]	Voretigene neparvovec	RPE65-IRD	BSC	£95,072/QALY	€114,033/QALY	Yes
Arjun Bhadhuri et al. 2022; Switzerland [73]	Voretigene neparvovec	RPE65-IRD	SoC	CHF 113,526/QALY	€70,813/QALY	No

All CE profiles (yes/no) are based on the threshold set in the original reference. *ICER chosen values response after 12 months from receiving therapy by Amy Gye, et al. 2022; **ICER values selected were those referred to the longest horizon reported by Chihiro Yamamoto et al. 2024. Abbreviations: NR = not reported; Standard of care (SoC); incremental cost-effectiveness ratio (ICER); quality-adjusted life-year (QALY); Life-years (LYs); United States (US);C$ = Canadian dollars; A$ = Australian dollarsJoshua A. Roth et al; 2018; US; chemotherapy = rituximab, dexamethasone,cytarabine, and cisplatin ; Jee H. Choe et al, 2022; US: SoC = Salvage chemotherapy followed by hematopoietic stem cell trans-plantation; Christopher Hillis et al, 2022; Canada: BSC: chemotherapy options including gemcitabine, etoposide and cyclophosphamide; Kambhampati et al, 2022; US; SoC = salvage chemoimmunotherapy followed by autologous stem cell transplant; Swetha Miguel-Angel Perales et al;2022; US; SoC = salvage chemoimmunotherapy, followed by high-dose therapy with autologous stem cells; Anne sofie Ledgaard Loftager et al ; Sweden; SoC = platinum-based chemoimmunotherapy followed by consolidation with highdose therapy and autologous stem cell transplantation; SoC = Blinatumomab; Na li et al, 2023; US and China; rituximab, ifosfamide, carboplatin, and etoposide [R-ICE]; rituximab, gemcitabine, dexamethasone, and cisplatin (R-GDP); rituximab, dexamethasone, cytarabine,and cisplatin (R-DHAP); and rituximab,etoposide, cytarabine, and cisplatin (R-ESHAP); Olalekan O. Oluwole et al,2023; US; SoC = chemotherapy; Alejandro Martín García-Sancho et al, 2024; Spain; SoC = salvage immunochemotherapy followed by high-dose chemotherapy and autologous stem-cell transplantation ; Saaya Tsutsué et al; 2024, Japan ; salvage chemoimmunotherapy, followed by high-dose therapy with autologous stem cell rescue for responders:; Olalekan O. Oluwole et al, 2024; US; SoC = stem cell therapy and monoclonal antibody therapy; Reith R. Sarkar et al;2019; US; SoC = clofarabine, etoposide, cyclophosphamide, followed by hematopoietic stem cell transplantation; Jill Furzer et al, 2020; Canada; SoC = intensive combination chemotherapy; Josep Maria Ribera Santasusana et al, 2020; Spain; salvage chemotherapy = combination of fludarabine, cytarabine, and idarubicin;Maziar Moradi-laken et al, 2021; Switzerland: ALL SoC = fludarabine, cytarabine, and idarubicin, clofarabine combination therapy (clofarabine, cyclophosphamide, and etoposide), and blinatumomab; DLBCL SoC = rituximab and anthracycline; Amy Gye et al, 2023; Australia = Blinatumomab, Amy Gye et al, 2024; Australia; SoC = Blinatumomab; Amy Gye et al, 2024; Australia; SoC = Blinatumomab ; Boon Piang Cher et al, 2020; Singapore; SoC = Association of rituximab, cyclophosphamide, doxorubicin, vincristine, prednisone; radiotherapy; association of stem cell transplant; association of rituximab, ifosfamide, carboplatin, etoposide, mesna, and peg-filgrastim; association of rituximab, dexamethasone, cytarabine, cisplatin, and peg-filgrastim; rituximab, gemcitabine, dexamethasone, and cisplatin; association of carmustine, etoposide, cytarabine, melphalan; Shiho Wakase et al, 2021, Japan: SoC = rituximab, ifosfamide, carboplatin, and etoposide; rituximab, gemcitabine, dexamethasone, and cisplatin; rituximab, etoposide, methylprednisolone, cytarabine, and cisplatin; rituximab, dexamethasone, cytarabine, and cisplatin (R-DHAP); and rituximab, etoposide, prednisolone, vincristine, cyclophosphamide, and doxorubixin ; Cynthia Z. Qi et al, 2021; US; SoC = salvage chemotherapy; Niamh Carey et al, 2023; Ireland; SoC: rituximab, gemcitabine, dexamethasone, cisplatin; with or without Hematopoietic stem cell transplantation; Lisa Masucci et al, 2024; Canada; SoC = rituximab, gemcitabine, dexamethasone, and cisplatin; Korinna Karampampa et al, 2023; Canada and France; SoC = arfilzomib-dexamethasone , dexamethasone pomalidomide, carfilzomib-dexamethasone-pomalidomide, daratumumab-dexamethasone-pomalidomide , and carfil-zomib-cyclophosphamide-dexamethasone; Claire L. Simons et al 2022; US: SoC = cytotoxic chemotherapy [bendamustine], proteasome inhibitors (bortezomib), immunomodulatory drugs (lenalidomide), Bcl-2 protein inhibitors (venetoclax), and BTK inhibitors (acalabrutinib, ibrutinib, Zanubrutinib) ; Graeme Ball et al, 2022; Canada = chemoimmunotherapy, as bendamustine and rituximab, rituximab and cyclophosphamide and doxorubicin and vincristine and prednisone; Svenja Petersohn et al, 2022, UK; SoC = citotox chemotherapy; M. Marchettia et al, 2023, Italy; SoC = Rituximab, bendamustine, cy-tarabine; Niklaus Meier et al, 2024; Germany; SoC = coagulation factor IX; Marita Zimmermann et al; 2019 ; US; SoC = regular physician visits and supportive care; Arjun Bhadhuri et al, 2022; Switzerland; best supportive care = such as visual aids, technical aids, support, and care in daily life.

Table S6. Key features of economic methods for the overall included studies.

Study References, Year	Country	Type of Economic Evaluation	Study design	Type of costs included	Perspective	Threshold (WTP)	Time horizon	Discount rate (base case)	Reference year of costs	Sensitivity analysis
Joshua A. Roth et al; 2018 [19]	US	Cost-effectiveness	Decision-tree model	Direct cost	Payer	$100,000 / QALY.	Lifetime	3%	2017	One-way and probabilistic
Melanie D. Whittington et al; 2018 [43]	US	Cost-effectiveness	Decision tree, Semi-Markov partitioned survival model	Direct cost	Payer	$100000/ QALY	Lifetime	3%	2017	Probabilistic sensitivity
Marita Zimmermann et al; 2019 [66]	US	Cost-Utility	Markov model	Direct costs	NHS	$ 250,000/QALY	Lifetime	3%	2017	One-way and probabilistic
Melanie D. Whittington et al; 2019 [21]	US	Cost-effectiveness	Long-term Survival Decision analytic model	Direct cost	Public payer and commercial payer	Not reported	Lifetime	/	Not reported	no
Reith R. Sarkar et al;2019 [44]	US	Cost-effectiveness	Microsimulatio n model	Direct cost	Third-party payer perspective	$200 000/QALY	Lifetime	3%	2017	One-way and probabilistic
Scott Johnson et al, 2019 [58]	US	Cost-effectiveness	Modeled Patient Population	Direct cost	NHS	$150 000/QALY	Lifetime	3%	2018	Deterministic probabilistic sensitivity
Daniel C. Malone et al, 2019 [62]	US	Cost-effectiveness analysis	Markov model	Direct cost	Commercial payer	$150,000 or $500,000/QALY	Lifetime	3%	Not reported	Deterministic and probabilistic
Frederick W. Thielen et al, 2020 [45]	The Netherlands	Cost-effectiveness analysis	Three-state partitioned survival model	Direct and indirect costs	NHS/society	€ 80,000/QALY	Lifetime	4%	Not reported	Deterministic and probabilistic
Jill Furzer at al, 2020 [78]	Canada	Cost-utility analysis	Three-state partitioned survival model	Direct cost	Public insurer	$ 150,000/QALY	Lifetime	1.5%	2018	Probabilistic
Boon Piang Cher et al, 2020 [71]	Singapore	Cost-effectiveness analysis	Hybrid decision tree and three-state partitioned survival model	Direct cost	NHS	S$200K/QALY	15-year	3%	2018	One-way and probabilistic
Matthias Fritz Uhrmann et al; 2020 [59]	Germany	Cost-effectiveness	Markov model	Direct and indirect costs	NHS/society	£300,000 / QALY	Lifetime	3%	Not reported	Not specified
Daniel Viriato et al; 2020 [60]	UK	Cost-effectiveness	Markov model	Direct cost	NHS	£100,000 / QALY	Lifetime	1.5%	2019	Probabilistic and univariate

Study References, Year	Country	Type of Economic Evaluation	Study design	Type of costs included	Perspective	Threshold (WTP)	Time horizon	Discount rate (base case)	Reference year of costs	Sensitivity analysis
Josep Maria Ribera Santasusana et al, 2020 [46]	Spain	Cost-Effectiveness and Cost-Utility	Partitioned survival model	Direct cost	NHS	€ 50,000/QALY	Lifetime	3%	2018	Deterministic
Shiho Wakase at al, 2021 [47]	Japan	Cost-effectiveness	Partitioned survival model	Direct cost	Healthcare payer	¥7.5million/QALY	Lifetime	2%	2018	Deterministic and probabilistic
Thomas F. Broekhoff, 2021 [68]	The Netherlands	Cost-effectiveness analysis	Individual-based state-transition model	Direct cost and indirect	Society	€ 80,000/QALY	Lifetime	4%	2019	Deterministic and probabilistic
Rongzhe Liu et al, 2021 [20]	US	Cost-effectiveness	Three-state partitioned survival model	Direct cost	Payer	$31,500/QALY	Lifetime	3%	2019	Univariate, probabilistic
Maziar Moradi-laken et al, 2021[48]	Switzerland	Cost-effectiveness and Budget Impact Analyses Cost-utility	Three-state partitioned survival model	Direct cost	NHS/society	CHF 150,000/QALY	Lifetime	3.5%	2021	Deterministic, probabilistic
Shiho Wakase et al, 2021 [79]	Japan	Cost-effectiveness analysis	Three-state partitioned survival model	Direct cost	Healthcare payer	¥7.5million/QALY	Lifetime	2%	2018	Deterministic and probabilistic
Rebecca Dean et al, 2021 [77]	US	Cost-utility	CUR model	Direct cost	Commercial payer	$150,000/QALY	Lifetime	3%	2019	no specified
Xiao Jun Wang et al, 2021 [49]	Singapore	Cost-effectiveness analysis Budget Impact Analyses	Three-health state partitioned survival model	Direct cost	Private insurance payer	S$266,973/QALY	Lifetime	3%	2020	Deterministic and probabilistic
Cynthia Z. Qi et al, 2021[70]	US	Cost-effectiveness and value of information analysis	Three-state responder-based partitioned survival model	Direct cost	Third-party payer	$150,000/QALY	Lifetime	3%	2020	Deterministic and probabilistic

Study References, Year	Country	Type of Economic Evaluation	Study design	Type of costs included	Perspective	Threshold (WTP)	Time horizon	Discount rate (base case)	Reference year of costs	Sensitivity analysis
Claire L. Simons et al 2021[54]	US	Cost-Effectiveness	Three-state partitioned survival model	Direct cost	Payer	$150,000/QALY	Lifetime	3%	2020	Deterministic and probabilistic
Xiao Jun Wang et al, 2022[69]	Singapore	Cost-effectiveness analysis Budget Impact Analyses	Three-health state partitioned survival model	Direct cost	NHS	S$266,973/QALY	Lifetime	3%	2020	Deterministic and probabilistic
Niamh Carey et al, 2022 [63]	Ireland	Cost-effectiveness and Budget Impact Analyses	Three-state partitioned survival model	Direct cost	Healthcare payer	€ 45,000/QALY	Lifetime	4%	2020	One-way
Olalekan O. Oluwole et al, 2022[22]	US	Cost-effectiveness	Three-state partitioned survival model	Direct cost	Payer	$150,000/QALY	Lifetime	3%	2021	Univariate and probabilistic
Graeme Ball et al, 2022 [50]	Canada	Cost-effectiveness Cost-utility	Three-health state, partitioned survival model	Direct cost	NHS	CAD 100,000 /QALY	Lifetime	1.5%	2021	One way, probabilistic,
Arjun Bhadhuri et al, 2022 [73]	Switzerland	Cost-effectiveness	Markov model	Direct and indirect costs	NHS/society	CHF 100,000 / QALY	Lifetime	3%	2018	Univariate and probabilistic
Jee H. Choe et al, 2022 [23]	US	Cost-effectiveness analysis	Three-state partitioned survival model	Direct costs	NHS	$150 000/QALY	Lifetime	3%	2021	Univariate and bayesian multivariate probabilistic
Alice Kate Cummings Joyner et al, 2022 [24]	US	Cost-effectiveness analysis	Decision-tree model	Direct cost	Payer	$150,000 /QALY	Lifetime	3%	2020	Deterministic and probabilistic
Christopher Hillis et al, 2022 [25]	Canada	Cost Effectiveness	Partitioned survival model, Partitioned Survival Mixture Cure Model, Survival Estimates	Direct costs	NHS	$150,000/QALY	Lifetime	1.5%	2021	Univariate and Probabilistic Sensitivity Analysis

Study References, Year	Country	Type of Economic Evaluation	Study design	Type of costs included	Perspective	Threshold (WTP)	Time horizon	Discount rate (base case)	Reference year of costs	Sensitivity analysis
Svenja Petersohn et al, 2022[51]	UK	Cost-effectiveness	Three-state partitioned survival model	Direct cost	NHS	£80 000/ QALY	Lifetime	3,5	2021	Probabilistic sensitivity
Bijal D. Shah et al, 2022 [53]	US	Cost-Effectiveness	Decision-tree, partitioned survival analysis	Direct cost	third party payer	$150,000/QALY	Lifetime	3%	2021	univariate and probabilistic
Swetha Kambhampati et al, 2022 [26]	US	Cost-effectiveness analysis	State-transition Markov model	Direct cost	NHS	$150 000/QALY	Lifetime	3%	2021	One-way and probabilistic
Miguel-Angel Perales et al;2022 [27]	US	Cost-effectiveness	Three-state partitioned survival model	Direct cost	Third-party payer	$150,000 /QALY,	Lifetime	3%	2021	One-way and probabilistic
Na li et al, 2022 [28]	China	Cost-effectiveness analysis	Short-term decision tree and long-term semi-Markov partitioned survival model	Direct cost	NHS	$31,320/QALY	Lifetime	5%	2020	Univariate and probabilistic
Mariana Baston-Oreiro et al, 2022 [29]	Spain	Cost-effectiveness, cost-utility analysis	Partitioned survival mixture cure model	Direct cost	NHS	€ 22,000/QALY or € 60,000/QALY	Lifetime	3%	2020	One-way and probabilistic
Tianjiao Wang et al; 2022 [76]	Australia	Cost-effectiveness	Markov model	Direct cost	NHS	$50,000 / QALY	Lifetime	5%	2020	One-way
Amy Gye et al, 2022 [72]	Australia	Cost-effectiveness	Partitioned survival model	Direct cost	NHS	USD 98,450/ QALY	Lifetime	5%	2022	no specified
Weijia Wu et al, 2023 [30]	China	Cost-effectiveness analysis	Markov model	Direct cost	NHS	CNY 257,241/QALY	Lifetime	5%	2022	One-way and probabilistic
Kandice A. Kapinos et al, 2023 [56]	US	Cost-Effectiveness	Monte Carlo Markov Chain microsimulatio n model	Direct cost	Healthcare payer	$100,000/QALY	20 months	3%	2020	Probabilistic

Study References, Year	Country	Type of Economic Evaluation	Study design	Type of costs included	Perspective	Threshold (WTP)	Time horizon	Discount rate (base case)	Reference year of costs	Sensitivity analysis
Weijia Wu et al; 2023 [55]	China	Cost-effectiveness	Markov model	Direct cost	NHS	$37,65/QALY	Lifetime	5%	2021	One-way and probabilistic
Niamh Carey et al, 2023 [64]	Ireland	Cost utility and value of information analysis	Short-term decision tree and Three-state partitioned survival model	Direct cost	Healthcare payer	€45,000/QALY	Lifetime	4%	2020	One-way
Anne sofie Ledgaard Loftager et al, 2023 [31]	Sweden	Cost-effectiveness analysis	Three-state partitioned survival model	Direct cost	NHS	SEK 1,000,000/QALY	Lifetime	3%	2022	Deterministic and probabilistic
Korinna Karampampa et al, 2023 [74]	France and Canada	Cost-effectiveness	Three-state partitioned survival model	Direct and indirect cost	society	Not reported:	Lifetime	France:2.5% Canada: 1.5%	2021	One-way deterministic sensitivity and probabilistic sensitivity analyses
M. Marchetti et al, 2023 [52]	Italy	Cost-Effectiveness	Partitioned-survival model	Direct cost	NHS	€87,330/ QALY	Lifetime	3%	2022	Univariate and probabilistic sensitivity
Christopher Parker et al, 2023 [75]	US	Cost-effectiveness	Three-state partitioned survival model	Direct cost	Third-party payer	$100,000/ QALY	Lifetime	3%	2020	Deterministic probabilistic sensitivity
Kunal C. Potnis et al, 2023 [39]	US	Cost-effectiveness	Markov model	Direct cost	Payer	$ 150,000 / QALY	Lifetime	3%	2021	Probabilistic sensitivity,
Na li et al, 2023 [32]	US and China	Cost-effectiveness	Tree and a long-term semi-markov partitioned survival model	Direct cost	NHS	USA $150,000/QALY China $37,654/ QALY	40 years	US: 3% China: 5%	2021	One-way and probabilistic
Amy Gye et al, 2024 [65]	Australia	Cost-effectiveness	Long-term modeled	Direct cost	NHS	USD 98,450/ QALY	Lifetime	5%	Not reported	One-way sensitivity
Alejandro Martín García-Sancho et al, 2024 [34]	Spain	Cost-Effectiveness	Cost-effectiveness model	Direct cost	NHS	EUR 60,000 / QALY	Lifetime	3%	2022	Deterministic and probabilistic sensitivity

Study References, Year	Country	Type of Economic Evaluation	Study design	Type of costs included	Perspective	Threshold (WTP)	Time horizon	Discount rate (base case)	Reference year of costs	Sensitivity analysis
Lisa Masucci et al, 2024 [61]	Canada	Cost-Utility	Individual-simulated discrete event simulation model	Direct cost	Healthcare Payer	$100,000/QALY,	Lifetime	1.5%	2021	One-way and probabilistic
Olalekan O. Oluwole et al,2023 [33]	US	Cost-effectiveness	Three-state partitioned survival model	Direct cost	Healthcare Payer	$150,000 / QALY	Lifetime	3%	2023	Deterministic probabilistic sensitivity
Niklaus Meier et al, 2024 [42]	Germany	Cost-Effectiveness	Microsimulation model	Direct cost	Healthcare Payer	€50,000/QALY	Lifetime	3%	2022	Univariate Sensitivity
Olalekan O. Oluwole et al, 2024 [35]	US	Cost-effectiveness	Three-state partitioned survival model	Direct cost	Healthcare payer	$150,000 / QALY	Lifetime	3%	2023	One-way and probabilistic
Olalekan O. Oluwole et al, 2024 [40]	US	Cost-effectiveness	Three-state partitioned survival model	Direct cost	Third-party payer	$150,000 / QALY	Lifetime	3%	2022	Deterministic probabilistic sensitivity
Olalekan O. Oluwole et al, 2024 [41]	US	Cost-effectiveness	Three-state partitioned survival model	Direct cost	Third-party payer	$150,000 / QALY	Lifetime	3%	2023	One-way and Probabilistic
Markqayne Ray et al ; 2024 [36]	France	Cost-effectiveness	Three-state partitioned survival model	Direct cost	NHS	European threshold	Lifetime	2.5%	2022	One way, probabilistic
Saaya Tsutsué et al; 2024 [37]	Japan	Cost-effectiveness	Three-state partitioned survival model	Direct cost	Payer	¥7.5million/QALY	Lifetime	2%	2023	One-way and probabilistic
Saaya Tsutsué et al ; 2024 [38]	Japan	Cost-effectiveness	Three-state partitioned survival model	Direct cost	Paye	¥7,500,000/ QALY	Lifetime	2%	2023	One-way and probabilistic
Chihiro Yamamoto et al, 2024 [57]	Japan, US	Cost-Effectiveness	Markov model	Direct cost	Payer	Japan: ¥7.5million/QALY USA: $150000/QALY	10 years	3%	2023	Deterministic and probabilistic
Amy Gye et al, 2024 [67]	Australia	Cost-Effectiveness	state-transition model, partitioned survival model, and discrete event simulation	Direct cost	NHS	Not reported	Lifetime	5%	2022	Deterministic

Table S7. Assessment of risk of bias and quality of evidence (from Olry de Labry-Lima et al., 2023) [10]

Domain	Item	Explanation
Design	Population	Does the study describe the eligible population?
	Interventions and comparators	Does the study describe the experimental intervention and comparator treatments?
	Study design	Is the study's design adequate for the decision problem (sample size, design of trials…)?
	Structural assumptions (models)	Are the assumptions implied by the design of the models described and reasonable?
	Time horizon	Does the study use an appropriate time horizon?
	Study perspective	Does the study state the viewpoint of the analysis and perform it correctly?
Data and analysis	Resources	Are the data for resource use disaggregated from costs?
	Unit costs	Are the unit costs reported?
	Outcomes	Are the outcomes included relevant?
	Discounting	Are costs and outcomes discounted, if appropriate?
	Main measure of effect	Does the study specify the main relevant effects (utilities, health benefits or other outcomes)?
	Sensitivity analyses	Does the study describe the analyses of uncertainty (bootstrapping, sensitivity analysis…)?
Interpretation	Authors' conclusions	Do the authors base their conclusions on the evidence?
Transferability	Transferability or generalizability	Are the research study’s findings applicable to other contexts, studies and populations?
Conflict of interest	Conflict of interest / ethical issues	Is there any potential conflict of interest?

Table S8. Quality appraisal of individual included studies according to the Checklist Olry de Labry-Lima et al., 2023

Study, year, country; reference	Quality appraisal via Checklist from Olry de Labry-Lima et al., 2023		Potential Risk of bias	Conflict of interest
	Scoring	Appraisal		
Joshua A. Roth et al; 2018 ; US [19]	Moderate/High ✔✔✔✔⊖	The effect estimate as the true effect is likely to be close to the estimate of the effect.	Uncertainty Bias, Study design Bias	Yes
Melanie D. Whittington et al; 2018; US [43]	Moderate/High ✔✔✔✔⊖	The effect estimate as the true effect is likely to be close to the estimate of the effect.	Selection Bias, Assumption Bias, Uncertainty Bias	No
Marita Zimmermann et al; 2019, US [66]	Moderate/High ✔✔✔✔⊖	The effect estimate as the true effect is likely to be close to the estimate of the effect.	Selection bias	No
Melanie D. Whittington et al; 2019; US [21]	Moderate/Low ✔✔ ⊖⊖⊖	The effect estimate as the true effect is likely to be close to the estimate of the effect, but there is a possibility that it is substantially different.	Selection bias, Assumption Bias	Yes
Reith R. Sarkar et al;2019; US [44]	Moderate ✔✔✔⊖⊖	The effect estimate as the true effect is likely to be close to the estimate of the effect, but there is a possibility that it is quite different.	Data availability bias, Selection bias, Uncertainty Bias	No
Scott Johnson et al, 2019; US [58]	Moderate/High ✔✔✔✔⊖	The effect estimate as the true effect is likely to be close to the estimate of the effect.	Selection bias, Assumption Bias	Yes
Daniel C. Malone et al, 2019; US [62]	Moderate ✔✔✔⊖⊖	The effect estimate as the true effect is likely to be close to the estimate of the effect, but there is a possibility that it is quite different.	Extrapolation bias, Study design Bias	Yes
Frederick W. Thielen et al, 2020; The Netherlands [45]	Moderate/High ✔✔✔✔⊖	The effect estimate as the true effect is likely to be close to the estimate of the effect.	Assumption Bias	Yes
Jill Furzer at al, 2020; Canada [78]	High ✔✔✔✔✔	The true effect lies close to that of the estimated effect.	Selection Bias	Yes
Boon Piang Cher et al, 2020; Singapore	Moderate ✔✔✔⊖⊖	The effect estimate as the true effect is likely to be close to the estimate of the effect, but there is a possibility that it is quite different.	Selection Bias, Uncertainty Bias, Data Availability Bias	Yes
Matthias Fritz Uhrmann et al; 2020, Germany [59]	Moderate ✔✔✔⊖⊖	The effect estimate as the true effect is likely to be close to the estimate of the effect, but there is a possibility that it is quite different.	Data Availability Bias, Underestimation Bias	No
Daniel Viriato et al; 2020, UK [60]	Moderate/High ✔✔✔✔⊖	The effect estimate as the true effect is likely to be close to the estimate of the effect.	Uncertainty bias	Yes
Josep Maria Ribera Santasusana et al, 2020; Spain [46]	High ✔✔✔✔✔	The true effect lies close to that of the estimated effect.	Extrapolation Bias, Assumption Bias	Yes
Shiho Wakase at al, 2021; Japan [47]	Moderate ✔✔✔⊖⊖	The effect estimate as the true effect is likely to be close to the estimate of the effect, but there is a possibility that it is quite different.	Extrapolation Bias	Yes

Study, year, country; reference	Quality appraisal via Checklist from Olry de Labry-Lima et al., 2023		Potential Risk of bias	Conflict of interest
	Scoring	Appraisal		
Thomas F. Broekhoff, 2021; Netherlands [68]	Moderate/High ✔✔✔✔⊖	The effect estimate as the true effect is likely to be close to the estimate of the effect.	Assumption bias, Selection bias,	No
Rongzhe Liu et al, 2021; US [20]	Moderate ✔✔✔⊖⊖	The effect estimate as the true effect is likely to be close to the estimate of the effect, but there is a possibility that it is quite different.	Selection Bias, Assumption Bias	No
Maziar Moradi-laken et al, 2021; Switzerland [48]	Moderate/High ✔✔✔✔⊖	The effect estimate as the true effect is likely to be close to the estimate of the effect.	Data Source Bias, Assumption Bias	Yes
Shiho Wakase et al, 2021, Japan [79]	Moderate ✔✔✔⊖⊖	The effect estimate as the true effect is likely to be close to the estimate of the effect, but there is a possibility that it is quite different.	Selection bias, Assumption bias, Data source bias	No
Rebecca Dean et al, 2021; US [77]	Moderate/High ✔✔✔✔⊖	The effect estimate as the true effect is likely to be close to the estimate of the effect.	Extrapolation Bias, Modelling Bias, Assumption Bias	Yes
Xiao Jun Wang et al, 2021; Singapore [49]	Moderate/High ✔✔✔✔⊖	The effect estimate as the true effect is likely to be close to the estimate of the effect.	Study design, Selection Bias,	Yes
Cynthia Z. Qi et al, 2021; US [70]	Moderate ✔✔✔⊖⊖	The effect estimate as the true effect is likely to be close to the estimate of the effect, but there is a possibility that it is quite different.	Data Availability Bias, Data source Bias, Study design Bias	Yes
Claire L. Simons et al 2022 ; US [54]	Moderate/High ✔✔✔✔⊖	The effect estimate as the true effect is likely to be close to the estimate of the effect.	Extrapolation Bias, Study design Bias, Data source Bias	Yes
Xiao Jun Wang et al, 2022; Singapore [69]	Moderate/High ✔✔✔✔⊖	The effect estimate as the true effect is likely to be close to the estimate of the effect.	Assumption Bias, Study design Bias	Yes
Niamh Carey et al, 2022; Ireland [63]	Moderate/High ✔✔✔✔⊖	The effect estimate as the true effect is likely to be close to the estimate of the effect.	Assumption bias, Modelling Bias	No
Olalekan O. Oluwole et al, 2022; US [22]	Moderate ✔✔✔⊖⊖	The effect estimate as the true effect is likely to be close to the estimate of the effect, but there is a possibility that it is quite different.	Extrapolation bias, Selection Bias, Data source bias	Yes
Graeme Ball et al, 2022; Canada [50]	Moderate ✔✔✔⊖⊖	The effect estimate as the true effect is likely to be close to the estimate of the effect, but there is a possibility that it is quite different.	Selection Bias, Data Source Bias, Extrapolation Bias	No
Arjun Bhadhuri et al, 2022; Switzerland [73]	Moderate/High ✔✔✔✔⊖	The effect estimate as the true effect is likely to be close to the estimate of the effect.	Extrapolation Bias, Omission Bias, Measurement Bias	Yes

Study, year, country; reference	Quality appraisal via Checklist from Olry de Labry-Lima et al., 2023		Potential Risk of bias	Conflict of interest
	Scoring	Appraisal		
Jee H. Choe et al, 2022 ; US [23]	Moderate ✓✓✓⊖⊖	The effect estimate as the true effect is likely to be close to the estimate of the effect, but there is a possibility that it is quite different.	Data Source Bias, Misclassification Bias	No
Alice Kate Cummings Joyner et al, 2022; US [24]	Moderate✓✓⊖⊖	The effect estimate as the true effect is likely to be close to the estimate of the effect, but there is a possibility that it is quite different.	Data Source Bias, Selection Bias, Measurement Bias	Yes
Christopher Hillis et al, 2022 ; Canada [25]	Moderate/High ✓✓✓✓⊖	The effect estimate as the true effect is likely to be close to the estimate of the effect.	Selection Bias, Data Availability Bias, Extrapolation Bias	Yes
Svenja Petersohn et al, 2022, UK [51]	Moderate/High ✓✓✓✓⊖	The effect estimate as the true effect is likely to be close to the estimate of the effect.	Extrapolation Bias,Selection Bias, Data Source Bias	Yes
Bijal D. Shah et al, 2022 US [53]	Moderate/High ✓✓✓✓⊖	The effect estimate as the true effect is likely to be close to the estimate of the effect.	Selection bias, Assumption bias, Extrapolation bias, Measurement Bias, Data Availability Bias	Yes
Swetha Kambhampati et al, 2022; US [26]	Moderate/High ✓✓✓✓⊖	The effect estimate as the true effect is likely to be close to the estimate of the effect.	Data Availability Bias, Extrapolation Bias, Assumption Bias	No
Miguel-Angel Perales et al;2022 ; US [27]	Moderate/High ✓✓✓✓⊖	The effect estimate as the true effect is likely to be close to the estimate of the effect.	Data Source Bias, Extrapolation Bias, Assumption Bias	No
Na li et al, 2022; China [28]	Moderate/High ✓✓✓✓⊖	The effect estimate as the true effect is likely to be close to the estimate of the effect.	Selection bias, Data Source Bias, Assumption Bias	Yes
Mariana Baston-Oreiro et al, 2022; Spain [29]	Moderate ✓✓✓⊖⊖	The effect estimate as the true effect is likely to be close to the estimate of the effect, but there is a possibility that it is quite different.	Study design Bias, Selection Bias, Data Availability Bias, Extrapolation Bias	No

Study, year, country; reference	Quality appraisal via Checklist from Olry de Labry-Lima et al., 2023		Potential Risk of bias	Conflict of interest
	Scoring	Appraisal		
Tianjiao Wang et al; 2022, Australia [76]	Moderate ✔✔✔⊖⊖	The effect estimate as the true effect is likely to be close to the estimate of the effect, but there is a possibility that it is quite different.	Modelling Bias	No
Amy Gye et al, 2022; Australia [72]	Moderate/High ✔✔✔✔⊖	The effect estimate as the true effect is likely to be close to the estimate of the effect.	Extrapolation bias	No
Weijia Wu et al, 2023; China [30]	Moderate/High ✔✔✔✔⊖	The effect estimate as the true effect is likely to be close to the estimate of the effect.	Data Source Bias, Selection Bias	Yes
Kandice A. Kapinos et al, 2023 ; US [56]	Moderate/High ✔✔✔✔⊖	The effect estimate as the true effect is likely to be close to the estimate of the effect.	Omission Bias, Study Design Bias	No
Weijia Wu et al; 2023 ; China [55]	Moderate ✔✔✔⊖⊖	The effect estimate as the true effect is likely to be close to the estimate of the effect, but there is a possibility that it is quite different.	Data Source Bias, Selection Bias	No
Niamh Carey et al, 2023 ; Ireland [64]	Moderate/High ✔✔✔✔⊖	The effect estimate as the true effect is likely to be close to the estimate of the effect.	Assumption Bias, Extrapolation Bias, Study Design Bias	No
Anne sofie Ledgaard Loftager et al, 2023 ; Sweden [31]	Moderate/High ✔✔✔✔⊖	The effect estimate as the true effect is likely to be close to the estimate of the effect.	Extrapolation Bias, Assumption Bias, Data Source Bias	Yes
Korinna Karampampa et al, 2023; Canada and France [74]	Moderate ✔✔✔⊖`⊖	The effect estimate as the true effect is likely to be close to the estimate of the effect, but there is a possibility that it is quite different.	Extrapolation Bias, Study Design Bias, Omission Bias, Assumption Bias	Yes
M. Marchetti et al, 2023, Italy [52]	Moderate/High ✔✔✔✔⊖	The effect estimate as the true effect is likely to be close to the estimate of the effect.	Study design bias, Assumption Bias, Extrapolation Bias	No
Christopher Parker et al, 2023; US [75]	Moderate/High ✔✔✔✔⊖	The effect estimate as the true effect is likely to be close to the estimate of the effect.	Data Availability Bias, Measurement Bias, Study Design Bias,Assumption Bias	No
Kunal C. Potnis et al, 2023; US[39]	Moderate/High ✔✔✔✔⊖	The effect estimate as the true effect is likely to be close to the estimate of the effect.	Study Design Bias, Extrapolation Bias, Measurement Bias	No

Study, year, country; reference	Quality appraisal via Checklist from Olry de Labry-Lima et al., 2023		Potential Risk of bias	Conflict of interest
	Scoring	Appraisal		
Na li et al, 2023; US and China [32]	Moderate/High ✔✔✔✔⊖	The effect estimate as the true effect is likely to be close to the estimate of the effect.	Measurement Bias, Model Bias, Assumption Bias	No
Amy Gye et al, 2024; Australia [65]	Moderate ✔✔✔⊖⊖	The effect estimate as the true effect is likely to be close to the estimate of the effect, but there is a possibility that it is quite different	Study Design Bias, Measurement Bias, Data Availability Bias	Yes
Alejandro Martín García-Sancho et al, 2024, Spain [34]	Moderate/High ✔✔✔✔⊖	The effect estimate as the true effect is likely to be close to the estimate of the effect.	Omission Bias, Study Design Bias	No
Lisa Masucci et al, 2024; Canada [61]	Moderate ✔✔✔⊖⊖	The effect estimate as the true effect is likely to be close to the estimate of the effect, but there is a possibility that it is quite different.	Study Design Bias, Extrapolation Bias, Modeling Bias	No
Olalekan O. Oluwole et al,2023 ; US [33]	High ✔✔✔✔✔	The true effect lies close to that of the estimated effect.	Data Availability Bias	Yes
Niklaus Meier et al, 2024; Germany[42]	Moderate ✔✔✔⊖⊖	The effect estimate as the true effect is likely to be close to the estimate of the effect, but there is a possibility that it is quite different.	Data Availability Bias, Assumption Bias, Model Bias	No
Olalekan O. Oluwole et al, 2024 ; US [35]	Moderate/High ✔✔✔✔⊖	The effect estimate as the true effect is likely to be close to the estimate of the effect.	Study Design Bias, Modelling Bias, Data Availability Bias	No
Olalekan O. Oluwole et al, 2024; US [40]	High ✔✔✔✔✔	The true effect lies close to that of the estimated effect.	Study design Bias	Yes
Olalekan O. Oluwole et al, 2024; US [41]	High ✔✔✔✔✔	The true effect lies close to that of the estimated effect.	Study design Bias, Modelling Bias	Yes
Markqayne Ray et al ; 2024 ; France [36]	High ✔✔✔✔✔	The true effect lies close to that of the estimated effect.	Modelling Bias	Yes
Saaya Tsutsué et al; 2024 ; Japan [37]	Moderate/High ✔✔✔✔⊖	The effect estimate as the true effect is likely to be close to the estimate of the effect.	Extrapolation Bias	Yes
Saaya Tsutsué et al ; 2024 ; Japan [38]	Moderate ✔✔✔⊖⊖	The effect estimate as the true effect is likely to be close to the estimate of the effect, but there is a possibility that it is quite different.	Data avaibility Bias, Extrapolation Bias	Yes
Chihiro Yamamoto et al. 2024, Japan and US [57]	High ✔✔✔✔✔	The true effect lies close to that of the estimated effect.	Assumptions Bias	No
Amy Gye et al, 2024, Australia [67]	Moderate/High ✔✔✔✔⊖	The effect estimate as the true effect is likely to be close to the estimate of the effect.	Data Availability Bias	Yes
